# Comprehensive analyses of circulating cardiometabolic proteins and objective measures of fat mass

**DOI:** 10.1038/s41366-023-01351-z

**Published:** 2023-08-07

**Authors:** Olga E. Titova, Carl Brunius, Eva Warensjö Lemming, Karl Stattin, John A. Baron, Liisa Byberg, Karl Michaëlsson, Susanna C. Larsson

**Affiliations:** 1 Unit of Medical Epidemiology, Department of Surgical Sciences, Uppsala University, Uppsala, Sweden; 2 Department of Biology and Biological Engineering, Food and Nutrition Science, Chalmers University of Technology, Gothenburg, Sweden; 3 Department of Food studies, nutrition and dietetics, Uppsala University, Uppsala, Sweden; 4 Department of Surgical Sciences, Anaesthesiology and Intensive Care, Uppsala University, Uppsala, Sweden; 5 Department of Medicine, University of North Carolina School of Medicine, Chapel Hill, NC USA; 6 Department of Epidemiology, Gillings School of Global Public Health, University of North Carolina, Chapel Hill, NC USA; 7 Unit of Cardiovascular and Nutritional Epidemiology, Institute of Environmental Medicine, Karolinska Institutet, Stockholm, Sweden

**Keywords:** Risk factors, Medical research

## Abstract

**Background:**

The underlying molecular pathways for the effect of excess fat mass on cardiometabolic diseases is not well understood. Since body mass index is a suboptimal measure of body fat content, we investigated the relationship of fat mass measured by dual-energy X-ray absorptiometry with circulating cardiometabolic proteins.

**Methods:**

We used data from a population-based cohort of 4950 Swedish women (55–85 years), divided into discovery and replication samples; 276 proteins were assessed with three Olink Proseek Multiplex panels. We used random forest to identify the most relevant biomarker candidates related to fat mass index (FMI), multivariable linear regression to further investigate the associations between FMI characteristics and circulating proteins adjusted for potential confounders, and principal component analysis (PCA) for the detection of common covariance patterns among the proteins.

**Results:**

Total FMI was associated with 66 proteins following adjustment for multiple testing in discovery and replication multivariable analyses. Five proteins not previously associated with body size were associated with either lower FMI (calsyntenin-2 (CLSTN2), kallikrein-10 (KLK10)), or higher FMI (scavenger receptor cysteine-rich domain-containing group B protein (SSC4D), trem-like transcript 2 protein (TLT-2), and interleukin-6 receptor subunit alpha (IL-6RA)). PCA provided an efficient summary of the main variation in FMI-related circulating proteins involved in glucose and lipid metabolism, appetite regulation, adipocyte differentiation, immune response and inflammation. Similar patterns were observed for regional fat mass measures.

**Conclusions:**

This is the first large study showing associations between fat mass and circulating cardiometabolic proteins. Proteins not previously linked to body size are implicated in modulation of postsynaptic signals, inflammation, and carcinogenesis.

## Introduction

Obesity is a major modifiable risk factor for numerous adverse health outcomes such as cardiovascular diseases (CVDs), type 2 diabetes, several types of cancer, and mortality [1, 2]. The cardiovascular consequences of obesity may occur through several different mechanistic pathways, including atherosclerosis, hypertension, diabetes, thrombosis, and endothelial dysfunction [3]. However, the specific mechanisms are not well understood. Modern technologies, such as proteomics, provide new possibilities to simultaneously investigate a large number of circulating proteins with potential clinical relevance. Although common measures of body size, including body mass index (BMI), waist circumference (WC) and waist-to-hip ratio (WHR), have been linked to several cardiometabolic proteins [4–8], replication of these findings and identification of novel associations is needed. In a longitudinal study in the elderly, several proteins were associated with changes only in either BMI or WHR, indicating that associations between circulating proteins and various measures of body size and fat distribution may differ [7].

BMI is the most commonly used measure of adiposity, but it does not provide insight into the body fat distribution or tissue composition. Body composition scans allow the evaluation of fat mass independently of other tissue constituents [9]. A recent Mendelian randomization study found that the fat mass index (FMI) is a better predictor of risk of major cardiometabolic diseases than BMI [10]. However, there have been no studies of objectively measured fat mass in relation to cardiometabolic circulating proteins. Discovery and validation of proteins related to body composition are important for better understanding of the potential molecular pathways underlying the link between obesity and cardiometabolic diseases, and may suggest potential therapeutic targets.

In the present study, we investigated for the first time the associations between total and regional fat mass and 276 circulating cardiometabolic proteins. We employed a combination of machine learning and conventional statistical analysis to identify associations with high predictive value in a population-based cohort of 4950 Swedish women.

## Materials and methods

### Study population

We used a previously described clinical subcohort [11] of the population-based Swedish Mammography Cohort (SMC; www.simpler4health.se), which included participants from central Sweden. Between November 2003 and October 2009, the 5022 women who were randomly chosen for the subcohort underwent a clinical examination that included dual-energy X-ray absorptiometry (DXA) measurements, blood and urine samples, fat biopsies and anthropometric assessment. Previously, participants had responded to SMC lifestyle, food frequency and health questionnaires in 1987–1990, 1997, and up to one month before the clinical examination. From the initial sample size, we excluded for the present analysis 72 individuals because the protein profile was not measured (*n* = 25) or fat mass data was not obtained (*n* = 47). The study was approved by the Ethical Review Agency, Sweden. All participants gave written informed consent.

### Assessment of body composition and potential confounders

Body composition measurements were obtained with DXA (Lunar Prodigy; Lunar Corp, Madison, WI, USA). The same trained personnel and the same DXA equipment were used for all study participants. The precision errors on triple DXA scans in 15 participants, including repositioning, were 0.8–1.5% depending on type of measurement (lean mass, fat mass or bone mineral density) and site [12]. The long-term coefficient of variation was less than 1% for a spine phantom. The validity of fat mass measured by Lunar Prodigy has been evaluated against the 4-compartment model, the current gold standard for body composition appraisal, resulting in 1.7–2.0% higher fat mass estimates with use of DXA [12]. Total fat mass was approximated using the fat mass index (FMI), calculated as total fat mass divided by the square of height (kg/m^2^). Android and gynoid fat mass was also expressed in terms of FMI (kg/m^2^), and named as android FMI and gynoid FMI, respectively. In addition, the ratio android/gynoid fat mass was calculated. Lifestyle information assessed by the questionnaires included participants’ educational attainment, walking/bicycling, leisure-time physical activity, alcohol consumption, and smoking status. The physical activity questionnaire has been validated using 7-day activity records and accelerometer data [13]. Weight (kg) and height (cm) were measured at the clinical examinations.

### Proteomic profiling

The proteomic analyses in this subcohort have been previously described [14]. Briefly, venous blood samples were collected after an overnight fast. Samples were immediately centrifuged and stored at −80 °C until analysis. Analysis of 276 protein biomarkers was performed utilizing three high-throughput multiplex immunoassays: the Olink Proseek Multiplex CVD II, CVD III, and Metabolism (Olink Bioscience, Uppsala, Sweden), each measuring 92 selected CVD or metabolism-related proteins simultaneously. In previous proteomic analysis based on the same cohort, no substantial systematic drift between analysis plates was detected [15]. Protein names and abbreviations can be found in the Supplementary Table 1. The analyses were performed at SciLifeLab, Uppsala University, Sweden. Inter-plate variability was adjusted for by intensity normalization with the plate median as the normalization factor. For data analysis Olink NPX Manager Software was used. The results provide relative values, normalized protein expression (NPX) data, which are log_2_ transformed; one-unit higher NPX represents an approximate doubling of the protein concentration. The PEA assays have mean intra-assay and inter-assay coefficients of variation around 8 and 12%, respectively. Values below the limit of detection (LOD) were used as provided by the manufacturer. Fourteen proteins with more than 75% of samples below the LOD were excluded from the analysis in accordance with the manufacturer’s recommendations (Supplementary Table 2**)**. In the analysis of the CVD II, CVD III and Metabolism panels, respectively 147 (3.0% of 4950 analyzed specimens), 8 (0.2%), and 7 (0.1%) samples did not pass the manufacturer’s quality control and were therefore set to missing. N-terminal prohormone of brain natriuretic peptide (NT-proBNP) was measured in both the CVD III and Metabolism panels. The latter was used for the analyses since more values passed the internal quality controls in that panel. The remaining 261 protein values were standardized (mean = 0, and standard deviation = 1) in order to obtain comparable estimates.

### Statistical analysis

Due to the expected large number of associations of fat mass with circulating proteins [5], we applied a random forest (RF)-based machine learning method with minimally biased variable selection to identify the most predictive associations, while minimizing overfitting and false positive findings [16]. To further adjust for potential confounders, we subsequently performed a multivariable analysis of FMI-protein associations and applied multiple comparison adjustment. We used a discovery (80%) and replication (20%) design, applied both to the machine learning and linear models (Supplementary Fig. 1). Statistical analyses were performed using Stata version 15.1 (StataCorp, College Station, TX, USA) or R software version 4.0.5 (R Core Team, Vienna, Austria), and the statistical tests were two-sided.

### Identification of proteins related to fat mass index using the Multivariate methods with Unbiased Variable selection (MUVR) algorithm

To explore associations between fat mass and the proteome, we first applied RF modeling with the measured proteome data as predictors and FMI as the target. RF modeling was conducted using the MUVR algorithm (v 0.0.975) [16] with the following parameters: number of repetitions (*nRep*) = 30, number of folds in the outer cross-validation loop (*nOuter*) = 6 and proportion of features kept per iteration in the recursive feature elimination in the inner cross-validation loop (*varRatio*) = 0.8. The MUVR algorithm utilizes repeated double cross validation to reduce overfitting and false positive discovery and performs a minimally biased variable selection to provide an optimal selection of proteins associated with FMI [16]. The variable selection is guided by the performance of successively fewer variables (determined by *varRatio*) on calibration data within the double cross validation, resulting in a best selection of predictor variables based on modeling performance. The predictive performance for the MUVR-RF regression was calculated as Q2 = 1 – PRESS /TSS, where PRESS is the prediction error sums of squares and TSS is the total sums of squares. Consequently, Q2 is interpreted similarly to R^2^, with Q2 = 1 representing perfect prediction and Q2 = 0 representing a null regression.

### Discovery and replication of proteins linked to fat mass characteristics using multivariable regression analyses

In the discovery sample (*n* = 3960), separate multiple linear regression analyses were performed for each protein selected from the MUVR algorithm, with protein as an outcome (dependent) variable and FMI as an exposure (independent) variable. Potential confounders were selected using directed acyclic graphs (DAGs) [17] based on our a priori knowledge of the relationships among potential confounders, intermediate variables, exposure, and outcome variables, as well as on existing information regarding factors associated with fat mass or BMI and circulating proteins [18, 19]. The analyses were adjusted for age (continuous), educational attainment (≤9 years, 10–12 years or >12 years of school), alcohol intake (g/day, the product of frequency of consumption of beer, wine, and liquor and amounts consumed at each occasion), current smoking status (no/yes), walking/cycling (hardly/ever; <20 min/day; 20–40 min/day; 40–60 min/day; 60–90 min/day; and >90 min/day), leisure-time exercise (<1 h/week; 1 h/week; 2–3 h/week; 4–5 h/week; and >5 hours/week) and lean mass. To account for multiple testing, a 5% False Discovery Rate (FDR) using the Benjamini and Hochberg procedure was applied [20]. Among 4,950 participants, 14% had missing information in leisure-time exercise, and 13% on walking/bicycling habits. Information on education, and alcohol intake was missing in less than 1%. Missing information on covariates was imputed using multiple imputation with chained equations and 20 imputation cycles. Proteins were measured using the same technology and analyzed using the same scale, therefore correlations among proteins were estimated using Pearson correlations.

Proteins associated with FMI at a 5% FDR in the discovery sample were assessed in the replication cohort using multiple regression analyses with the same adjustments. We considered the association of FMI and individual proteins to be “replicated” if the nominal P value in the replication cohort was <0.05, and the direction of the association was the same. Finally, to obtain more accurate estimates for replicated proteins, we reperformed the analyses using the entire cohort.

Sensitivity analyses based on entire cohort were performed investigating the association of android, gynoid FMI as well as the android/gynoid fat mass ratio with proteins that were linked to total FMI (in the discovery and replication samples) using multivariable linear regression adjusted for potentially confounding variables as in the analysis of total FMI. In order to obtain more comparable estimates in this analysis, android, gynoid and total FMI we standardized setting the mean to zero and a standard deviation to one.

### Principal component analysis

In the third step, to obtain an overview of proteins and their associations with total FMI, we performed a ‘Varimax’ (orthogonal) rotated principal component analysis (PCA). FMI (exposure) was then associated with the component scores (outcomes) using multivariable linear regression adjusted for potentially confounding variables as in the step 2 above. Protein patterns and associations with FMI for the components were then visualized using the TriPlot algorithm (v 0.1.4) [21].

## Results

Baseline characteristics of the study participants are described in Table 1. Briefly, the mean age was 67.6 (SD 6.8) years and the mean FMI was 10.1 (3.2) kg/m^2^; 60% of the women had excess fat (FMI > 9 kg/m^2^) and 4% had a fat deficit (FMI < 5 kg/m^2^) according to the ranges of FMI that match the prevalence of the WHO BMI classifications for women [22]. Baseline characteristics of women randomly allocated to the discovery or replication samples are shown in the Supplementary table 3.

**Table 1:** Baseline characteristics of the participants.

Characteristics*	
Number of participants	4950
Age, years	67.6 (6.8)
Education, *n* (%)	
* ≤9 years*	1183 (24.0)
* 10–12 years*	1907 (38.6)
* >12 years*	1848 (37.4)
Current cigarette smoking status, *n* (%)	
* Non-smokers*	4509 (91.1)
* Current smokers*	441 (8.9)
Alcohol intake, g/day	6.2 (7.1)
Walking/bicycling, *n* (%)	
* Hardly ever*	431 (10.1)
* <20* *min/day*	635 (14.8)
* 20–40* *min/day*	1504 (35.1)
* 40–60* *min/day*	981 (22.9)
* 60–90* *min/day*	480 (11.2)
* >90* *min/day*	257 (6.0)
Exercise, *n* (%)	
* <1* *hour/week*	810 (19.0)
* 1* *h/week*	879 (20.6)
* 2–3* *h/week*	1415 (33.2)
* 4–5* *h/week*	613 (14.4)
* ≥5* *h/week*	541 (12.7)
Body mass index, kg/m^2^	25.9 (4.1)
Total fat mass, kg	26.9 (8.7)
Android fat mass, kg	2.3 (1.0)
Gynoid fat mass, kg	4.9 (1.3)
Android/gynoid fat mass ratio	0.5 (0.1)
Total lean mass, kg	39.4 (4.4)
Fat mass index, kg/m^2^	10.1 (3.2)

^*^Values are means (SD) or n (percentages).

In the first step of the data analysis, FMI showed a strong association with the measured proteome, as assessed by the MUVR-RF analysis. Predictive performance was high both in the discovery data (Q2 = 0.72) and in the replication data (Q2 = 0.73), suggesting strong FMI-proteome associations as well as the absence of overfitting or bias during model training (Supplementary Fig. 2). Of the 261 proteins which were initially included in the analysis, MUVR identified 105 as robustly linked to FMI (Supplementary Table 4). In the second step, in the discovery sample, FMI was associated with 80 proteins in multiple linear regression analyses adjusted for confounders and multiple comparisons. FMI was associated with 66 of these proteins in the replication sample.

**Fig. 1:**
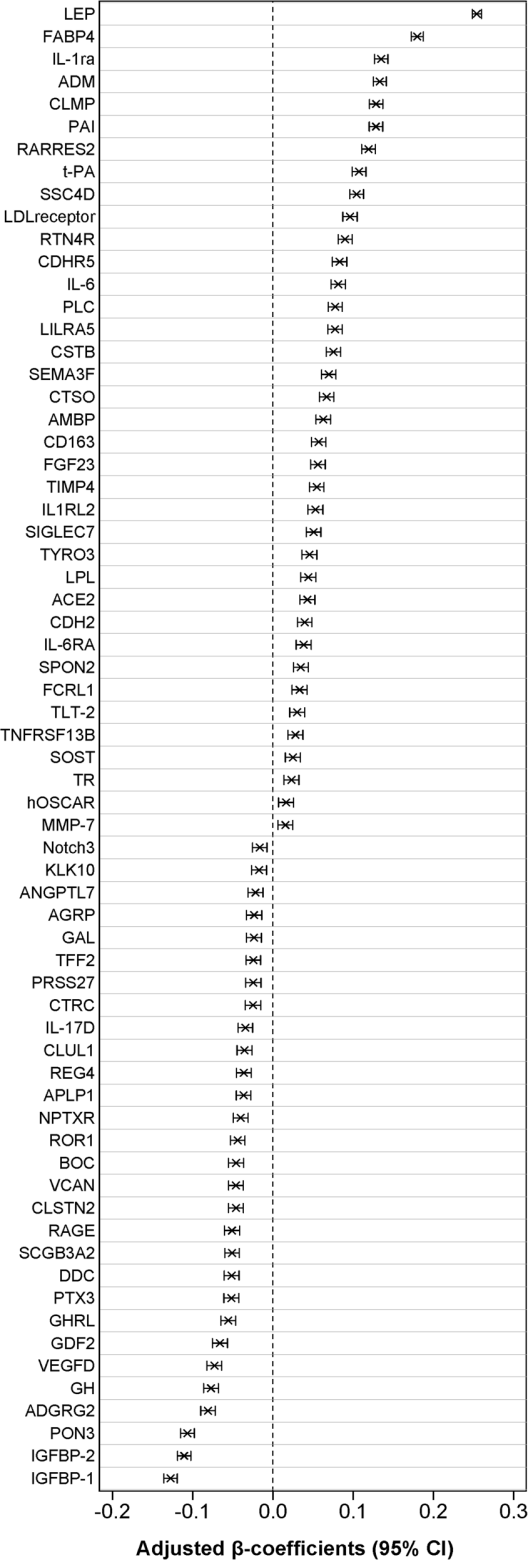
Association between fat mass index and circulating protein biomarkers. β-estimates (per 1 standard deviation change in biomarker concentration) and 95% CI derived from multiple linear regression analyses. The models were adjusted for age, educational attainment, alcohol intake, smoking status, walking/cycling, leisure-time exercise, and lean mass. The complete names of the abbreviated proteins can be found in Supplementary Table 1.

For these replicated 66 proteins, the analysis using the entire cohort is shown in Fig. 1 and Supplementary table 4. Higher FMI was associated with higher levels of 37 proteins; the strongest associations were with leptin (LEP), fatty acid-binding protein adipocyte (FABP4), adrenomedullin (ADM), interleukin-1 receptor antagonist protein (IL-1ra), CXADR-like membrane protein (CLMP), plasminogen activator inhibitor 1 (PAI), retinoic acid receptor responder protein 2 (RARRES2), tissue-type plasminogen activator (t-PA), scavenger receptor cysteine-rich domain-containing group B protein (SSC4D), and low-density lipoprotein receptor (LDL receptor). β-coefficients for these proteins in the regression analyses with FMI ranged from 0.016 (for matrix metalloproteinase-7, MMP-7, and osteoclast-associated immunoglobulin-like receptor, hOSCAR) to 0.254 for LEP (Fig. 1 and Supplementary Table 4). Higher FMI was associated with lower levels of 29 proteins, such as insulin-like growth factor-binding protein 1 (IGFBP-1), insulin-like growth factor-binding protein 2 (IGFBP-2), paraoxonase (PON3), adhesion G-protein coupled receptor G2 (ADGRG2), growth hormone (GH), vascular endothelial growth factor D (VEGF-D), and growth differentiation factor 2 (GDF2). β-coefficients in the regression analyses of these proteins ranged from −0.016 for neurogenic locus notch homolog protein 3 (Notch3) to −0.128 for IGFBP-1 (Fig. 1 and Supplementary Table 4). The correlation matrix of the 105 proteins identified in the first step is shown in Supplementary Fig. 3. Similar association patterns were observed for android and gynoid FMI for the most proteins. However, gynoid FMI was not linked to matrix metalloproteinase-7 (MMP-7) and Neurogenic locus notch homolog protein 3 (Notch3) (Supplementary Fig. 4). The association of the android/gynoid fat mass ratio with 66 proteins is shown in the Supplementary Fig. 5.

**Fig. 2:**
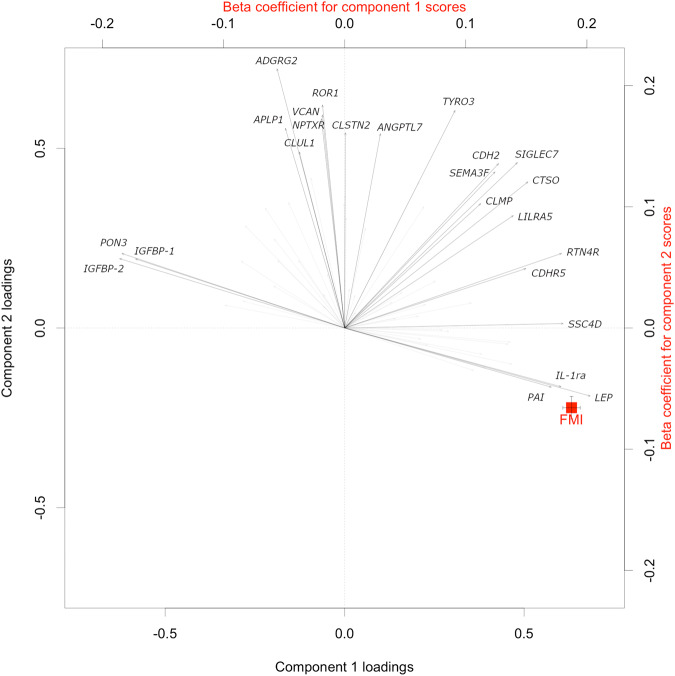
Associations of fat mass index (FMI) with circulating protein patterns. β-estimates (per 1 standard deviation change in principal component scores obtained from PCA of 66 selected proteins) and 95% CI derived from multiple linear regression analyses. The models were adjusted for age, educational attainment, alcohol intake, smoking status, walking/cycling, leisure-time exercise, and lean mass. For clarity, proteins with absolute loading values below 0.5 were grayed out (all loadings are reported in Supplementary Table 6). The complete names of the abbreviated proteins can be found in Supplementary Table 1.

The number of principal components was truncated to 4 according to the Velicer’s MAP (minimum average partial) criterion [23]. The 4 components explained 37% of the variance of the 66 proteins with confirmed associations with FMI. The first component showed a strong positive association with FMI (β-estimate = 0.187 per SD, *p* < 0.0001), with the strongest loadings from LEP, SSC4D, PAI, RTN4R, CDHR5, CTSO and IL-1ra (positive loadings), and IGFBP-2, IGFBP-1 and PON3 (negative loadings) (Fig. 2**and** Supplementary tables 5 and 6). Pearson correlation analysis revealed statistically significant associations among all these ten proteins with absolute value correlations of 0.18 ≤ |r | ≤ 0.63 (Supplementary Fig. 3). The three other principal components had statistically significant but less pronounced associations with FMI with the following β- estimates per SD: −0.066 (second principal component, PC), −0.011 (third PC), and 0.073 (fourth PC) (Fig. 2**and** Supplementary table 5).

## Discussion

Using a combined approach of RF-based analysis, linear models, and principal component analysis, the present study showed strong associations between FMI and multiple circulating proteins related to CVD and metabolism. We identified five proteins involved in carcinogenesis, postsynaptic signaling and inflammation that have not previously been related to body size measurements in a general population: scavenger receptor cysteine-rich domain-containing group B protein (SSC4D), calsyntenin-2 (CLSTN2), kallikrein-10 (KLK10), trem-like transcript 2 protein (TLT-2), and interleukin-6 receptor subunit alpha (IL-6RA). In addition, the PCA showed that FMI-associated proteins could be combined into components representing protein patterns, of which the first component had the strongest association with FMI. These proteins are involved in glucose and lipid metabolism, adipocyte differentiation, appetite regulation, immune response and inflammation.

Several studies have investigated the association of body size parameters, such as waist circumference, waist-to-hip ratio and BMI, with circulating proteins measured with multiplexed immunoassay targeted technologies (e.g., SOMALogic or Olink) [4, 5, 7, 8]. However, we are not aware of any study that has systematically examined the associations between objectively measured fat mass and a large number of proteins. Our study confirmed previously observed strong associations of different body size measurements with several circulating proteins involved in pathways such as triglyceride metabolism, appetite regulation, adipocyte differentiation, immune response, inflammation, hormone metabolism, and other biological processes [4, 5, 8]. In particular, we observed strong associations of elevated FMI with higher levels of multiple proteins, such as LEP, FABP4, IL-1ra, ADM, CLMP, PAI, RARRES2, t-PA, SSC4D, and LDL receptor, and lower concentrations of several other proteins, such as IGFBP-1, IGFBP-2, PON3, ADGRG2, and GH. Recent Mendelian randomization analyses suggested casual associations between several proteins identified and replicated in the present study. For example, a causal positive association of FABP4, LEP and PAI with BMI [8, 24], ADM with WHR [24], as well as inverse association of IGFBP-1, IGFBP-2, and GDF2 with BMI was suggested [8].

The PCA-based analysis in our study demonstrated that several proteins were captured in principal components that, in turn, associated with FMI. The protein pattern captured by the first component had the strongest association with FMI and the discussion here is therefore limited to the proteins loading most strongly in this component. These included LEP, SSC4D, PAI, reticulon-4 receptor (RTN4R), cadherin-related family member 5 (CDHR5), cathepsin O (CTSO) and IL-1ra (positive loadings), and insulin-like growth factor-binding protein 1 and 2 (IGFBP-1 and IGFBP-2) and paraoxonase (PON3) (negative loadings). These proteins tended to be correlated (positively or negatively) with each other, and individually associated with FMI. However, it should be noted that there were other proteins not captured by the first principal component that were associated with FMI, including FABP4, ADM, RARRES2, t-PA, and ADGRG2, suggesting other mechanisms.

The proteins with the highest loadings in principal component 1 are involved in several biological processes such as glucose and lipid metabolism, appetite regulation, adipocyte differentiation, immune response and inflammation. For instance, IGFBP-1 and IGFBP-2 which had inverse associations with FMI in our study, belong to the group of transport proteins involved in insulin-like growth factors (IGF) transport, bioavailability and function. They are thus implicated in glucose metabolism homeostasis [25, 26], which could explain why these proteins correlate highly (*r* = 0.63). The observed associations with FMI strengthens the previously reported inverse correlation of these proteins with BMI, fasting insulin levels and blood pressure [25, 27]. It is also suggested that the majority of IGFBPs have IGF-independent actions [25].

In our study, LEP had the strongest association with FMI, and was inversely associated with both IGFBP-1 and IGFBP-2 (*r* = −0.46 and −0.44, respectively). LEP is secreted by adipose tissue and plays an important role in the regulation of energy homeostasis, metabolism, and neuroendocrine and immune functions [28]. It has in fact been demonstrated both in vitro and in vivo that LEP regulates muscle expression of IGFBP-2, which could affect insulin sensitivity and glucose metabolism [29]. An inverse association with FMI was also observed for PON3. Evidence suggests that it is implicated in the pathophysiology of CVDs: PON3 can be bound to HDL in the bloodstream, and may decrease atherosclerosis progression as do other members of the PON family [30].

Further links to inflammation are supported by IL-1ra, which had a positive association with FMI. It belongs to the intrerleukin-1 (IL-1) cytokine family, inhibits the activity of IL-1-α and IL-1-β, and modulates immune and inflammatory responses related to IL-1 [31]. IL-1ra expression is induced by IL-1 and by other inflammatory stimuli [31]. SSC4D was also strongly linked to FMI. This protein belongs to the highly conserved scavenger receptor cysteine-rich (SRCR) superfamily of cell surface and/or secreted proteins, which relate to immune function [32]. Notably, SSC4D is not well described in the literature, and no previous studies have investigated an association with body size measurements.

In addition, we identified several other novel associations between FMI and circulating proteins, such as CLSTN2, KLK10, TLT-2, and IL-6RA. For example, CLSTN2 had a strong inverse association with FMI. This protein belongs to the calsyntenins family of postsynaptic membrane proteins, and may modulate calcium-mediated postsynaptic signals [33]. Previous human and animal studies have linked CLSTN2 alleles with cognitive performance [34–36]. However, the role of CLSTN2 is poorly characterized in relation to cellular function and molecular interactions [36], and has not been reported to be associated with metabolic health. KLK10 was inversely associated with FMI in our study. It is a member of the Kallikreins protein family, which is described in the literature mainly in relation to its role in carcinogenesis, although evidence is contradictory. For example, some studies have reported that KLK10 is elevated in pancreatic ductal adenocarcinoma tissues, and that aberrant KLK10 expression is associated with poor prognosis and shorter survival [37]. In contrast, KLK10 was also proposed to have a tumor-suppressor role in breast and prostate cancer [38]. In a previous cross-sectional study of 2444 participants, circulating KLK10 was not found to be associated with WC [4]. This may indicate that KLK10 is exclusively associated with FMI or that the divergent findings are related to technical differences in protein identification.

Among other novel identified associations, TLT-2 had a strong positive association with FMI. This finding was strengthened by a similar association observed with WC in another population-based study, although it did not retain statistical significance after adjustment for multiple comparisons [4]. In a randomized trial of dietary intervention in individuals with overweight/obesity, TLT-2 had a positive association with baseline BMI [6]. TLT-2 has previously been suggested to play a role in immune responses and inflammation [39] and experimental evidence suggests that TLT-2 is expressed in human monocytes and granulocytes and B cells, and that the expression can be upregulated in response to inflammatory mediators [39].

Further mechanistic links between adiposity and inflammation are suggested through the positive association between FMI and interleukin-6 receptor subunit alpha (IL-6RA) in our study. IL-6RA stimulates IL-6 activity and inflammatory responses [40, 41]. In a randomized trial of dietary intervention, proteomic profiles before and during weight loss was assessed in 609 adults with overweight/obesity. In this study, Il-6RA was not associated with BMI at baseline, but longitudinal analysis revealed a decrease in protein levels upon weight loss [6]. Taken together, the observed association of FMI with several inflammation-related proteins (e.g. IL-1ra, TLT-2 and IL-6RA) suggests possible mechanistic links between adiposity and inflammation. Extensive data suggests that inflammation plays an important role in atherosclerosis and CVDs [42].

In the present study, similar association patterns were observed for android and gynoid FMI for most of the proteins except MMP-7 and Notch3, which associated with android but not gynoid FMI. MMP-7 is involved in pathways controlling cell growth, inflammation, angiogenesis, and carcinogenesis [43]. Notch3, like other proteins in the Notch family, is implicated in regulating cell self-renewal, differentiation and plasticity of the vascular smooth muscle cells [44]. Animal studies in Notch3-deficient mice suggest that Notch3 plays an important role in coronary adaptation to pressure overload and further risk of heart failure [45]. Overexpression of Notch3 is also associated with cancer development [44]. The observed differential protein associations are in line with known differences in the relationship between android and gynoid fat with adverse health outcomes [46] and should be investigated in further studies.

Important strengths of our study are the large sample size, objectively and accurately assessed body composition, simultaneous measurements of a large number of proteins and the ability to adjust for important confounders. In addition, the replication analysis provided a robust evaluation of the associations between FMI and proteins. Moreover, we applied an RF algorithm with a stringent cross validation framework to limit the likelihood of overfitting and to provide an optimal selection of relevant proteins associated with FMI. Furthermore, the PCA-based analysis allowed us to get a holistic overview of associations between the measured proteome and FMI, while adjusting for confounders.

Several limitations, however, apply to this observational study. Proteins included in the Olink kits contain known cardiometabolic markers as well some human proteins which could be related to cardiovascular disease and metabolism. There is a possibility that other proteins not included in these panels could be associated more strongly or more meaningfully with FMI. This cohort included only women, and the results need to be verified in men. This study included Swedish adults only, and the generalizability of our results to other populations is unknown. Another limitation is the lack of an independent replication sample. Due to the observational nature of this study, we cannot rule out residual and unmeasured confounding. In addition, other health conditions could affect the observed association. Although we hypothesized that fat mass affects the levels of circulating proteins, it is difficult to prove the causal direction of the associations due to the cross-sectional design of our study.

In conclusion, in the present exploratory study of middle-aged and elderly women, for the first time, we investigated the association of fat mass with circulating proteins related to CVD and metabolism. Compared with other studies using proxy measures of fat mass (e.g., BMI or waist circumference), associations with five proteins were novel and 61 associations were similar to those related to other body size measurements. Furthermore, the group of proteins which had the strongest association with FMI are involved in glucose and lipid metabolism, appetite regulation, adipocyte differentiation, immune response and inflammation, suggesting potential pathways for the link between adiposity and risk of cardiometabolic disorders. Future studies should be directed to investigate how protein concentrations respond to changes in fat mass as well as the association of circulating proteins with subcutaneous and visceral fat. This could help to identify adiposity-related pathophysiological mechanisms which contribute to health deterioration including the development of CVDs.

### Supplementary information

Supplementary material

## Data Availability

The data that support findings of this study are available upon application to the Swedish Infrastructure for Medical Population-based Life-course Environmental Research (SIMPLER; https://www.simpler4health.se).
