# Supplementary material for: Comprehensive analyses of circulating cardiometabolic proteins and objective measures of fat mass
Source: Int J Obes (Lond). 2023 Aug 7;47(11):1043–9. doi: 10.1038/s41366-023-01351-z (PMC10599989; doi:10.1038/s41366-023-01351-z)

Supplementary Material

Comprehensive analyses of circulating cardiometabolic proteins and objective measures of fat mass

Olga E Titova, Carl Brunius, Eva Warensjö Lemming, Karl Stattin, John A Baron, Liisa Byberg, Karl Michaëlsson and Susanna C Larsson

Content: ***Page:***

Content:	Page:
**Supplementary Table 1.** The protein biomarkers included in the CVD II, CVD III and metabolism assay panels	2-7
**Supplementary Table 2.** Protein biomarkers excluded in the quality control in the CVD II, CVD III and metabolic panels	8
**Supplementary Table 3.** Baseline characteristics of women in the discovery and replication sub cohorts, SMC-C	9-10
**Supplementary Table 4.** Association of total fat mass index with the cardiometabolic proteins identified by the Multivariate methods with Unbiased Variable selection (MUVR) algorithm, total cohort.	11-15
**Supplementary Table 5.** Principal components (obtained by the PCA), and the association of these components with FMI (results derived from the multivariable linear regression analysis).	16
**Supplementary Table 6.** PCA loadings of 66 replicated proteins (Varimax rotation, 4 components).	17-18
**Supplementary Figure 1.** Flow chart and Study design	19
**Supplementary Figure 2.** Random Forest predictions of FMI from protein profiles using the MUVR algorithm for the discovery data used for model training (left) and the replication data never used for training (right).	20
**Supplementary Figure 3.** Correlation matrix of 105 protein biomarkers identified by the Multivariate methods with Unbiased Variable selection (MUVR).	21
**Supplementary Figure 4.** Association between total (A), android (B) and gynoid (C) fat mass index and circulating protein biomarkers.	22
**Supplementary Figure 5.** Association between *android/gynoid fat mass ratio* and circulating protein biomarkers.	23

Supplementary Table 1. The protein biomarkers included in the CVD II, CVD III and metabolism assay panels

CVD II panel		CVD III panel		Metabolism panel	
**Abbreviation**	**Name**	**Abbreviation**	**Name**	**Abbreviation**	**Name**
ACE2	Angiotensin-converting enzyme 2	ALCAM	CD166 antigen	AHCY	Adenosylhomocysteinase
ADAM-TS13	A disintegrin and metalloproteinase withthrombospondin motifs 13	AP-N	Aminopeptidase N	ADGRE2	Adhesion G protein-coupled receptor E2
ADM	Adrenomedullin	AXL	Tyrosine-protein kinase receptor UFO	ADGRG2	Adhesion G-protein coupled receptor G2
AGRP	Agouti-related protein	AZU1	Azurocidin	APLP1	Amyloid-like protein 1
AMBP	Protein AMBP	BLM hydrolase	Bleomycin hydrolase	ANGPT2	Angiopoietin-2
ANGPT1	Angiopoietin-1	CASP-3	Caspase-3	ANGPTL1	Angiopoietin-related protein 1
BMP-6	Bone morphogenetic protein 6	CCL15	C-C motif chemokine 15	ANGPTL7	Angiopoietin-related protein 7
BNP	Natriuretic peptides B	CCL16	C-C motif chemokine 16	ANXA11	Annexin A11
CA5A	Carbonic anhydrase 5A, mitochondrial	CCL22	C-C motif chemikine 22	ANXA4	Annexin A4
CCL17	C-C motif chemokine 17	CCL24	C-C motif chemokine 24	GHRL	Appetite-regulating hormone
CCL3	C-C motif chemokine 3	CD163	Scavenger receptor cysteine-rich type 1 protein M130	ARG1	Arginase-1
CD4	T-cell surface glycoprotein CD4	CD93	Complement component C1q receptor	DDC	Aromatic-L-amino-acid decarboxylase
CD40-L	CD40 ligand	CDH5	Cadherin-5	CD79B	B-cell antigen receptor complex-associated protein beta chain
CD84	SLAM family member 5	CHI3L1	Chitinase-3-like protein 1	CDH2	Cadherin-2

CEACAM8	Carcinoembryonic antigenrelated cell adhesion molecule 8	CHIT1	Chitotriosidase-1	CDHR5	Cadherin-related family member 5
CTRC	Chymotrypsin C	CNTN1	Contactin-1	CLSTN2	Calsyntenin-2
CTSL1	Cathepsin L1	COL1A1	Collagen alpha-1(I) chain	CA13	Carbonic anhydrase 13
CXCL1	C-X-C motif chemokine 1	CPA1	Carboxypeptidase A1	COMT	Catechol O-methyltransferase
DCN	Decorin	CPB1	Carboxypeptidase B	CTSO	Cathepsin O
DECR1	2,4-dienoyl-CoA reductase, mitochondrial	CSTB	Cystatin-B	CD2AP	CD2-associated protein
Dkk-1	Dickkopf-related protein 1	CTSD	Cathepsin D	CHRDL2	Chordin-like protein 2
FABP2	Fatty acid-binding protein, intestinal	CTSZ	Cathepsin Z	CLUL1	Clusterin-like protein 1
FGF21	Fibroblast growth factor 21	CXCL16	C-X-C motif chemokine 16	CCDC80	Coiled-coil domain-containing protein 80
FGF-23	Fibroblast growth factor 23	DLK-1	Protein delta homolog 1	CRKL	Crk-like protein
FS	Follistatin	EGFR	Epidermal growth factor receptor	CLEC5A	C-type lectin domain family 5 member A
Gal-9	Galectin-9	Ep-CAM	Epithelial cell adhesion molecule	CLMP	CXADR-like membrane protein
GDF2	Growth differentiation factor 2	EPHB4	Ephrin type-B receptor 4	DIABLO	Diablo homolog, mitochondrial
GH	Growth hormone	FABP4	Fatty acid-binding protein, adipocyte	QDPR	Dihydropteridine reductase
GIF	Gastric intrinsic factor	FAS	Tumor necrosis factor receptor superfamily member 6	DPP7	Dipeptidyl peptidase 2
GLO1	Lactoylglutathione lyase	Gal-3	Galectin-3	DAB2	Disabled homolog 2
GT	Gastrotropin	Gal-4	Galectin-4	APEX1	DNA-(apurinic or apyrimidinic site) lyase

HAOX1	Hydroxyacid oxidase 1	GDF15	Growth differentiation factor 15	ENTPD5	Ectonucleoside triphosphate diphosphohydrolase 5
HB-EGF	Proheparin-binding EGF-like growth factor	GRN	Granulins	ENPP7	Ectonucleotide pyrophosphatase/phosphodiesterase family member 7
HO-1	Heme oxygenase 1	ICAM-2	Intercellular adhesion molecule 2	RNASE3	Eosinophil cationic protein
hOSCAR	Osteoclast-associated immunoglobulin-like receptor	IGFBP-1	Insulin-like growth factor-binding protein 1	FCRL1	Fc receptor-like protein 1
HSP 27	Heat shock 27 kDa protein	IGFBP-2	Insulin-like Growth Factor-Binding Protein 2	FBP1	Fructose-1,6-bisphosphatase 1
IDUA	Alpha-L-iduronidase	IGFBP-7	Insulin-like growth factor-binding protein 7	GAL	Galanin peptides
IgG Fc receptor II-b	Low affinity immunoglobulin gamma Fc region receptor II-b	IL-17RA	Interleukin-17 receptor A	ENO2	Gamma-enolase
IL16	Pro-interleukin-16	IL-18BP	Interleukin-18-binding protein	GLRX	Glutaredoxin-1
IL-17D	Interleukin-17D	IL-1RT1	Interleukin-1 receptor type 1	GRAP2	GRB2-related adapter protein 2
IL-18	Interleukin-18	IL-1RT2	Interleukin-1 receptor type 2	HDGF	Hepatoma-derived growth factor
IL-1ra	Interleukin-1 receptor antagonist protein	IL2RA	Interleukin-2 receptor subunit alpha	ROR1	Inactive tyrosine-protein kinase transmembrane receptor ROR1
IL1RL2	Interleukin-1 receptor-like 2	IL-6RA	Interleukin-6 receptor subunit alpha	IGFBPL1	Insulin-like growth factor-binding protein-like 1
IL-27	Interleukin-27	ITGB2	Integrin beta-2	ITGB7	Integrin beta-7
IL-4RA	Interleukin-4 receptor subunit alpha	JAM-A	Junctional adhesion molecule A	KLK10	Kallikrein-10
IL-6	Interleukin-6	KLK6	Kallikrein-6	KYAT1	Kynurenine--oxoglutarate transaminase 1

ITGB1BP2	Melusin	LDL receptor	Low-density lipoprotein receptor	BAG6	Large proline-rich protein BAG6
KIM-1	Kidney injury molecule 1	LTBR	Lymphotoxin-beta receptor	LRIG1	Leucine-rich repeats and immunoglobulin-like domains protein 1
LEP	Leptin	MB	Myoglobin	LILRA5	Leukocyte immunoglobulin-like receptor subfamily A member 5
LOX-1	Lectin-like oxidized LDL receptor 1	MCP-1	Monocyte chemotactic protein 1	LRP11	Low-density lipoprotein receptor-related protein 11
LPL	Lipoprotein lipase	MEPE	Matrix extracellular phosphoglycoprotein	ACP6	Lysophosphatidic acid phosphatase type 6
MARCO	Macrophage receptor MARCO	MMP-2	Matrix metalloproteinase-2	MEP1B	Meprin A subunit beta
MERTK	Tyrosine-protein kinase Mer	MMP-3	Matrix metalloproteinase-3	METRNL	Meteorin-like protein
MMP-12	Matrix metalloproteinase-12	MMP-9	Matrix metalloproteinase-9	MCFD2	Multiple coagulation factor deficiency protein 2
MMP-7	Matrix metalloproteinase-7	MPO	Myeloperoxidase	NADK	NAD kinase
NEMO	NF-kappa-B essential modulator	Notch 3	Neurogenic locus notch homolog protein 3	NECTIN2	Nectin-2
PAPPA	Pappalysin-1	NT-pro BNP	N-terminal prohormone brain natriuretic peptide	NPDC1	Neural proliferation differentiation and control protein 1
PAR-1	Proteinase-activated receptor 1	OPG	Osteoprotegerin	NPTXR	Neuronal pentraxin receptor
PARP-1	Poly [ADP-ribose] polymerase 1	OPN	Osteopontin	NOMO1	Nodal modulator 1
PDGF subunit B	Platelet-derived growth factor subunit B	PAI	Plasminogen activator inhibitor 1	NT-proBNP	N-terminal prohormone of brain natriuretic peptide
PD-L2	Programmed cell death 1 ligand 2	PCSK9	Proprotein convertase subtilisin/kexin type 9	PILRB	Paired immunoglobulin-like type 2 receptor beta
PIgR	Polymeric immunoglobulin receptor	PDGF subunit A	Platelet-derived growth factor subunit A	FKBP4	Peptidyl-prolyl cis-trans isomerase FKBP4

PGF	Placenta growth factor	PECAM-1	Platelet endothelial cell adhesion molecule	PAG1	Phosphoprotein associated with glycosphingolipid- enriched microdomains 1
PRELP	Prolargin	PGLYRP1	Peptidoglycan recognition protein 1	CTSH	Pro-cathepsin H
Protein BOC	Brother of CDO	PI3	Elafin	FAM3C	Protein FAM3C
PRSS27	Serine protease 27	PLC	Perlecan	PPP1R2	Protein phosphatase inhibitor 2
PRSS8	Prostasin	PON3	Paraoxonase (PON 3)	S100P	Protein S100-P
PSGL-1	P-selectin glycoprotein ligand 1	PRTN3	Myeloblastin	REG4	Regenerating islet-derived protein 4
PTX3	Pentraxin-related protein PTX3	PSPD	Pulmonary surfactant-associated protein D	RTN4R	Reticulon-4 receptor
RAGE	Receptor for advanced glycosylation end products	RARRES2	Retinoic acid receptor responder protein 2	ALDH1A1	Retinal dehydrogenase 1
REN	Renin	RETN	Resistin	NQO2	Ribosyldihydronicotinamide dehydrogenase [quinone]
SCF	Stem cell factor	SCGB3A2	Secretoglobin family 3A member 2	SSC4D	Scavenger receptor cysteine-rich domain-containing group B protein
SERPINA12	Serpin A12	SELE	E-selectin	SOST	Sclerostin
SLAMF7	SLAM family member 7	SELP	P-selectin	SEMA3F	Semaphorin-3F
SOD2	Superoxide dismutase [Mn], mitochondrial	SHPS-1	Tyrosine-protein phosphatase non- receptor type substrate 1	SERPINB6	Serpin B6
SORT1	Sortilin	SPON1	Spondin-1	SERPINB8	Serpin B8
SPON2	Spondin-2	ST2	ST2 protein	SIGLEC7	Sialic acid-binding Ig-like lectin 7

SRC	Proto-oncogene tyrosine-protein kinase Src	TFF3	Trefoil factor 3	CD164	Sialomucin core protein 24
STK4	Serine/threonine-protein kinase 4	TFPI	Tissue factor pathway inhibitor	CANT1	Soluble calcium-activated nucleotidase 1
TF	Tissue factor	TIMP4	Metalloproteinase inhibitor 4	SUMF2	Sulfatase-modifying factor 2
TGM2	Protein-glutamine gamma-glutamyltransferase 2	TLT-2	Trem-like transcript 2 protein	SNAP23	Synaptosomal-associated protein 23
THBS2	Thrombospondin-2	TNF-R1	Tumor necrosis factor receptor 1	SDC4	Syndecan-4
THPO	Thrombopoietin	TNF-R2	Tumor necrosis factor receptor 2	CD1C	T-cell surface glycoprotein CD1c
TIE2	Angiopoietin-1 receptor	TNFRSF10C	Tumor necrosis factor receptor superfamily member 10C	THOP1	Thimet oligopeptidase
TM	Thrombomodulin	TNFRSF14	Tumor necrosis factor receptor superfamily member 14	TXNDC5	Thioredoxin domain-containing protein 5
TNFRSF10A	Tumor necrosis factor receptor superfamily member 10A	TNFSF13B	Tumor necrosis factor ligand superfamily member 13B	TYMP	Thymidine phosphorylase
TNFRSF11A	Tumor necrosis factor receptor superfamily member 11A	t-PA	Tissue-type plasminogen activator	TSHB	Thyrotropin subunit beta
TNFRSF13B	Tumor necrosis factor receptor superfamily member 13B	TR	Transferrin receptor protein 1	TFF2	Trefoil factor 2
TRAIL-R2	TNF-related apoptosis-inducing ligand receptor 2	TR-AP	Tartrate-resistant acid phosphatase type 5	TINAGL1	Tubulointerstitial nephritis antigen-like
VEGF-D	Vascular endothelial growth factor D	uPA	Urokinase-type plasminogen activator	TYRO3	Tyrosine-protein kinase receptor TYRO3
VSIG2	V-set and immunoglobulin domain-containing protein 2	U-PAR	Urokinase plasminogen activator surface receptor	USP8	Ubiquitin carboxyl-terminal hydrolase 8
XCL1	Lymphotactin	vWF	von Willebrand factor	VCAN	Versican core protein

Supplementary Table 2. Protein biomarkers excluded in the quality control in the CVD II, CVD III and Metabolism panels (more than 75% of samples were below LOD)

CVD II panel	CVD III panel	Metabolism panel	
PARP1	SPON1	AHCY	ITGB7
PAPPA	CCL22	S100P	NQO2
		DIABLO	ARG1
		ANXA4	USP8
		ANXA11	DAB2

Abbreviations of proteins are explained in **Supplementary Table 1**.

Supplementary Table 3. Baseline characteristics of women in the discovery and replication subcohorts

**Characteristics***	**Discovery**	**Replication**
**Number of participants**	3960	990
**Age**, years	67.6 (6.8)	67.6 (6.6)
**Education**, n (%)		
≤ 9 years	944 (24.0)	239 (24.2)
10-12 years	1,543 (39.1)	364 (36.8)
> 12 years	1,462 (37.0)	386 (39.0)
**Cigarette smoking status,** n (%)		
Non-smokers	3,600 (90.9)	909 (91.8)
Current smokers	360 (9.1)	81 (8.2)
**Alcohol intake**, g/day	6.2 (7.2)	6.3 (6.8)
**Walking/bicycling**, n (%)		
Hardly ever	350 (10.3)	81 (9.3)
<20 min/day	507 (14.9)	128 (14.6)
20–40 min/day	1,187 (34.8)	317 (36.2)
40-60 min/day	781 (22.9)	200 (22.9)
60-90 min/day	381 (11.2)	99 (11.3)
>90 min/day	207 (6.1)	50 (5.7)
**Exercise,** n (%)		
< 1 hour/week	642 (19.0)	168 (19.3)
1 hour/week	697 (20.6)	182 (20.9)
2-3 hours/week	1,122 (33.1)	293 (33.7)
4-5 hours/week	490 (14.5)	123 (14.1)

≥5 hours/week	437 (12.9)	104 (12.0)
**Body mass index**, kg/m^2	25.9 (4.1)	26.1 (4.3)
**Total fat mass,** kg	26.8 (8.6)	27.4 (8.9)
**Total lean mass**, kg	39.4 (4.4)	39.5 (4.4)
**Lean mass index,** kg/m^2	14.7 (1.4)	14.8 (1.4)
**Fat mass index,** kg/m^2	10.0 (3.2)	10.2 (3.3)

*Values are means ± SD or percentages.

Supplementary Table 4. Association of total fat mass index with the cardiometabolic proteins identified by the Multivariate methods with Unbiased Variable selection (MUVR) algorithm, total cohort.

	**Protein abbreviation**	**β-estimate**	**SE**	**P-value**
1	LEP	0.254203	0.002933	0.000
2	FABP4	0.179908	0.003841	0.000
3	ADM	0.133269	0.00411	1.00E-208
4	IL-1ra	0.134972	0.004306	2.00E-196
5	CLMP	0.128627	0.00426	6.00E-184
6	IGFBP-1	-0.12751	0.004236	7.00E-183
7	PAI	0.128452	0.004296	1.00E-180
8	RARRES2	0.119206	0.004339	1.00E-154
9	IGFBP-2	-0.11055	0.004267	8.00E-139
10	t-PA	0.107409	0.004369	6.00E-126
11	PON3	-0.10645	0.004344	3.00E-125
12	SSC4D	0.104377	0.004409	2.00E-117
13	LDL receptor	0.096127	0.004551	7.30E-95
14	RTN4R	0.089973	0.004445	1.40E-87
15	CDHR5	0.08309	0.004597	9.20E-71
16	IL-6	0.081292	0.004543	2.30E-69
17	PLC	0.077659	0.004425	6.20E-67
18	ADGRG2	-0.08091	0.004623	1.40E-66
19	LILRA5	0.077447	0.004578	1.90E-62
20	CSTB	0.075409	0.004503	3.10E-61
21	GH	-0.07711	0.004717	1.70E-58

22	VEGF-D	-0.07287	0.004716	1.30E-52
23	SEMA3F	0.069607	0.004602	1.50E-50
24	CTSO	0.067059	0.00456	6.10E-48
25	GDF2	-0.06597	0.004753	5.60E-43
26	AMBP	0.062792	0.004618	2.40E-41
27	CD163	0.057043	0.004611	1.20E-34
28	FGF-23	0.056086	0.004656	6.10E-33
29	GHRL	-0.05557	0.004661	2.50E-32
30	TIMP4	0.054694	0.004594	3.00E-32
31	SCGB3A2	-0.05097	0.00458	2.00E-28
32	IL1RL2	0.052941	0.004769	2.70E-28
33	SIGLEC7	0.050724	0.004639	1.70E-27
34	DDC	-0.05142	0.004712	2.00E-27
35	PTX3	-0.05175	0.004742	2.10E-27
36	RAGE	-0.05094	0.004832	1.10E-25
37	VCAN	-0.04609	0.004665	8.10E-23
38	CLSTN2	-0.04611	0.004751	4.50E-22
39	TYRO3	0.045481	0.004762	2.00E-21
40	ROR1	-0.04386	0.004612	2.90E-21
41	Protein BOC	-0.04591	0.00484	3.70E-21
42	LPL	0.044098	0.004848	1.40E-19
43	ACE2	0.042991	0.004744	1.80E-19
44	CDH2	0.039711	0.004588	6.60E-18
45	NPTXR	-0.04006	0.004708	2.30E-17

46	IL-6RA	0.038332	0.004788	1.50E-15
47	APLP1	-0.03656	0.004613	2.80E-15
48	REG4	-0.03627	0.004663	9.00E-15
49	IL-17D	-0.03432	0.004593	9.40E-14
50	CLUL1	-0.03532	0.004772	1.60E-13
51	SPON2	0.034836	0.004734	2.20E-13
52	FCRL1	0.033216	0.004771	3.80E-12
53	TLT-2	0.030275	0.004753	2.10E-10
54	TNFRSF13B	0.028111	0.004771	4.10E-09
55	TFF2	-0.02434	0.004589	1.20E-07
56	PGF	0.023865	0.004501	1.20E-07
57	SOST	0.024891	0.004809	2.40E-07
58	CTRC	-0.02477	0.004871	3.80E-07
59	PRSS27	-0.02437	0.004877	6.10E-07
60	GAL	-0.02366	0.004748	6.50E-07
61	TR	0.02309	0.004763	1.30E-06
62	AGRP	-0.02345	0.004879	1.60E-06
63	ANGPTL7	-0.02171	0.004757	5.10E-06
64	FAM3C	0.020903	0.004625	6.30E-06
65	KIM-1	0.019835	0.004651	2.00E-05
66	PSPD	-0.0195	0.004799	4.90E-05
67	Dkk-1	-0.01785	0.004906	2.80E-04
68	KLK10	-0.01725	0.004787	3.20E-04
69	Notch 3	-0.01635	0.004609	3.90E-04

70	hOSCAR	0.016377	0.004755	5.80E-04
71	MMP-7	0.015613	0.004696	8.90E-04
72	FABP2	-0.01575	0.004887	1.30E-03
73	ADAM-TS13	-0.01394	0.004901	4.50E-03
74	SCF	-0.01361	0.004819	4.70E-03
75	CLEC5A	-0.01256	0.004581	6.10E-03
76	CCL15	0.01296	0.004743	6.30E-03
77	ANGPT2	0.011817	0.00463	1.10E-02
78	IGFBP-7	-0.01201	0.004714	1.10E-02
79	PIgR	-0.01196	0.004884	1.40E-02
80	HB-EGF	-0.01055	0.004806	2.80E-02
81	MMP-3	0.009874	0.004707	3.60E-02
82	TFF3	-0.00979	0.00467	3.60E-02
83	CPB1	0.009361	0.004814	5.20E-02
84	GIF	-0.00876	0.004889	7.30E-02
85	SOD2	-0.00831	0.004892	9.00E-02
86	DCN	-0.00761	0.004604	9.80E-02
87	VSIG2	-0.00775	0.004756	1.00E-01
88	SERPINA12	-0.00762	0.004901	1.20E-01
89	MB	-0.00671	0.004631	1.50E-01
90	THBS2	0.006582	0.004884	1.80E-01
91	ST2	0.005793	0.004747	2.20E-01
92	DPP7	0.004585	0.004755	3.30E-01
93	DLK-1	0.004226	0.004738	3.70E-01

94	CPA1	-0.00408	0.004811	4.00E-01
95	KLK6	-0.00376	0.004875	4.40E-01
96	ANGPT1	-0.00368	0.004889	4.50E-01
97	SLAMF7	-0.00268	0.004874	5.80E-01
98	CHIT1	-0.00258	0.004769	5.90E-01
99	CCL17	-0.00168	0.00482	7.30E-01
100	Gal-4	0.001609	0.004754	7.30E-01
101	TF	-0.00151	0.004755	7.50E-01
102	TSHB	-0.00143	0.00482	7.70E-01
103	THPO	0.001013	0.004902	8.40E-01
104	ACP6	0.000711	0.004829	8.80E-01
105	MEPE	-0.00045	0.004791	9.20E-01

The models were adjusted for age, educational attainment, alcohol intake, smoking status, walking/cycling, leisure-time exercise, and lean mass. β-estimates (per 1 standard deviation change in biomarker concentration) and SE derived from the multiple linear regression analyses. The complete names of the abbreviated proteins can be found in **Supplementary Table 1**. SE, standard error

Supplementary Table 5. Principal components (obtained by the PCA), and the association of these components with FMI (results derived from the multivariable linear regression analysis).

**Results of the Principal Component Analysis (PCA)***		**Results from multivariable linear regression**	
**Component (VAF[a])**	***Loadings*** **Protein (loading value)**	**Beta**	**p**
1 (10.6%)	LEP (0.684); SSC4D (0.609); RTN4R (0.604) ; IL-1ra (0.603); PAI (0.576); CTSO (0.510); CDHR5 (0.505); IGFBP2 (-0.627); PON3 (-0.621); IGFBP1 (-0.583)	0.187	**0.00e+00**
2 (9.0%)	ADGRG2 (0.722); ROR1 (0.621); TYRO3 (0.606); VCAN (0.593); NPTXR (0.582); APLP1 (0.556); CLSTN2 (0.544); ANGPTL7 (0.541)	-0.066	**2.10e-43**
3 (8.9%)	SPON2 (0.768); AMBP (0.654); ADM (0.594); RAGE (0.563); hOSCAR (0.557); MMP-7 (0.542); TNFRSF13B (0.516); AGRP (0.506)	-0.011	**1.67e-02**
4 (8.5%)	PLC (0.764); RARRES2 (0.671); CSTB (0.651); Notch 3 (0.605); FABP4 (0.599); TLT-2 (0.580); IL-6RA (0.580); CD163 (0.561); TIMP4 (0.540)	0.073	**6.94e-58**

*****Arbitrary cutoff of loading value 0.5 was used, i.e., only proteins with loading values > 0.5 are shown in this table. The complete names of the abbreviated proteins can be found in **Supplementary Table 1**. The analysis was adjusted for the same covariates as in the main analysis.
[a] VAF, Variance Accounted For

Supplementary Table 6. PCA loadings of 66 replicated proteins (Varimax rotation, 4 components).

Protein abbreviation	*Component 1*	*Component 2*	*Component 3*	*Component 4*
LEP	0.684289973	-0.189608126	0.071816904	0.211940882
FABP4	0.466046907	-0.101054658	0.211056724	0.598740773
ADM	0.382301253	-0.073254788	0.593985486	0.347241617
IL-1ra	0.603012718	-0.163765601	0.22661944	0.264367767
CLMP	0.379142004	0.346325895	0.377404738	0.369776194
IGFBP-1	-0.58269425	0.192444676	0.288923082	0.243069506
PAI	0.575819696	-0.164733662	-0.028520868	0.251536919
RARRES2	0.391980908	-0.110761207	0.122529042	0.671192807
IGFBP-2	-0.627046534	0.193163066	0.305273959	0.375800078
t-PA	0.461606051	-0.039406782	0.046059107	0.471053888
PON3	-0.620783993	0.20755564	-0.131077626	0.141759714
SSC4D	0.609099897	0.0122916	-0.038371155	0.046129564
LDL receptor	0.455857347	-0.045109051	-0.026492999	0.34207488
RTN4R	0.604429963	0.207637143	0.173680017	0.148576021
CDHR5	0.505032543	0.165347386	-0.011982306	0.035151796
IL-6	0.359220397	-0.118382879	0.251025112	0.281121484
PLC	0.132054495	0.071461703	0.353241437	0.764339407
ADGRG2	-0.188575938	0.721988035	0.006213643	-0.082577205
LILRA5	0.470084258	0.312877743	0.181816064	0.271355298
CSTB	0.23178719	-0.047784013	0.22855664	0.650924388
GH	-0.333479027	0.063389739	0.133833546	-0.087449519
VEGF-D	-0.288415641	0.186130156	0.406266565	-0.110465016
SEMA3F	0.419032271	0.434604207	0.323307244	0.337865456
CTSO	0.510758879	0.406860036	0.193639802	0.172420397
GDF2	-0.22130142	0.333570351	0.303527356	-0.159885135
AMBP	0.272268544	-0.005186837	0.654256458	0.1599226
CD163	0.252926284	0.130260708	0.033827372	0.560891552
FGF23	0.290099992	-0.00926025	0.480090014	0.237498511
GHRL	-0.19658526	0.247653367	0.031112364	-0.085924698
TIMP4	-0.058542978	0.061031522	0.180431822	0.540286827
SCGB3A2	-0.283411976	0.075012484	0.155577247	0.211454459
IL1RL2	0.22539128	0.06504262	0.195195655	0.013820692
SIGLEC7	0.481414704	0.460552481	0.082711412	0.192268532
DDC	-0.094174889	0.417609862	-0.05142497	-0.087175132
PTX3	-0.19804545	0.117007798	0.295679916	0.060963062

RAGE	-0.186500904	0.188052271	0.563400842	0.036907257
VCAN	-0.062202861	0.593203861	0.187591433	0.191037081
CLSTN2	0.002056449	0.543628545	0.201107643	0.067141262
TYRO3	0.307815103	0.605536244	0.044835859	0.090406101
ROR1	-0.061864975	0.620881283	0.384597424	0.229803625
BOC	-0.156287344	0.348998125	0.421526415	-0.080171274
LPL	-0.059176695	0.093522291	0.360334011	0.116860985
ACE2	0.353149059	0.069594215	0.26793761	0.015937417
CDH2	0.429017583	0.457686371	0.252840883	0.193324414
NPTXR	-0.064812193	0.582408008	0.156568541	0.10772921
IL-6RA	0.030500197	0.043013798	-0.030585149	0.5799974
APLP1	-0.165446865	0.55581935	0.050468494	0.00396669
REG4	0.004157903	0.306626189	0.331960134	0.155917243
IL-17D	-0.128848251	0.18758812	0.457123422	0.11067848
CLUL1	-0.126628319	0.490304596	0.032122439	0.01591236
SPON2	0.205799436	0.032921291	0.766774372	0.151030715
FCRL1	0.22098362	0.337733074	0.04223729	0.177902003
TLT-2	0.028824994	0.090173097	0.105687809	0.580119925
TNFRSF13B	0.141685539	0.026492965	0.51630743	0.204286469
TFF2	0.057768705	0.277481738	0.317531481	0.140810364
SOST	0.207842637	0.405725206	0.062498479	0.076567312
CTRC	0.000513713	0.135022479	0.143284787	-0.115674542
PRSS27	-0.047755159	0.149013307	0.42425217	0.012007843
GAL	-0.001485474	0.345514905	-0.001122449	-0.086133701
TR	0.062685172	0.063441739	0.064599209	0.387793423
AGRP	0.012636435	0.1082996	0.506326865	0.026702917
ANGPTL7	0.099756355	0.540992343	0.248293755	0.058996231
KLK10	0.000935025	0.368132537	0.249495266	0.119738734
Notch3	-0.277063521	0.284115459	0.134927208	0.605023945
hOSCAR	0.160505263	0.098922586	0.556567148	0.18526599
MMP-7	0.212082333	-0.030793502	0.542444173	0.088143934

The complete names of the abbreviated proteins can be found in **Supplementary Table 1**.

Supplementary Figure 1. Flow chart and Study design. [a] MUVR-RF **-** a random forest based Multivariate methods with Unbiased Variable selection; [b] Of the initial 276 proteins, fourteen proteins with more than 75% of samples below the limit of detection (LOD) were excluded from the analysis; NT-proBNP was measured in the CVD III and Metabolism panels, and only the latter was used for the analyses.

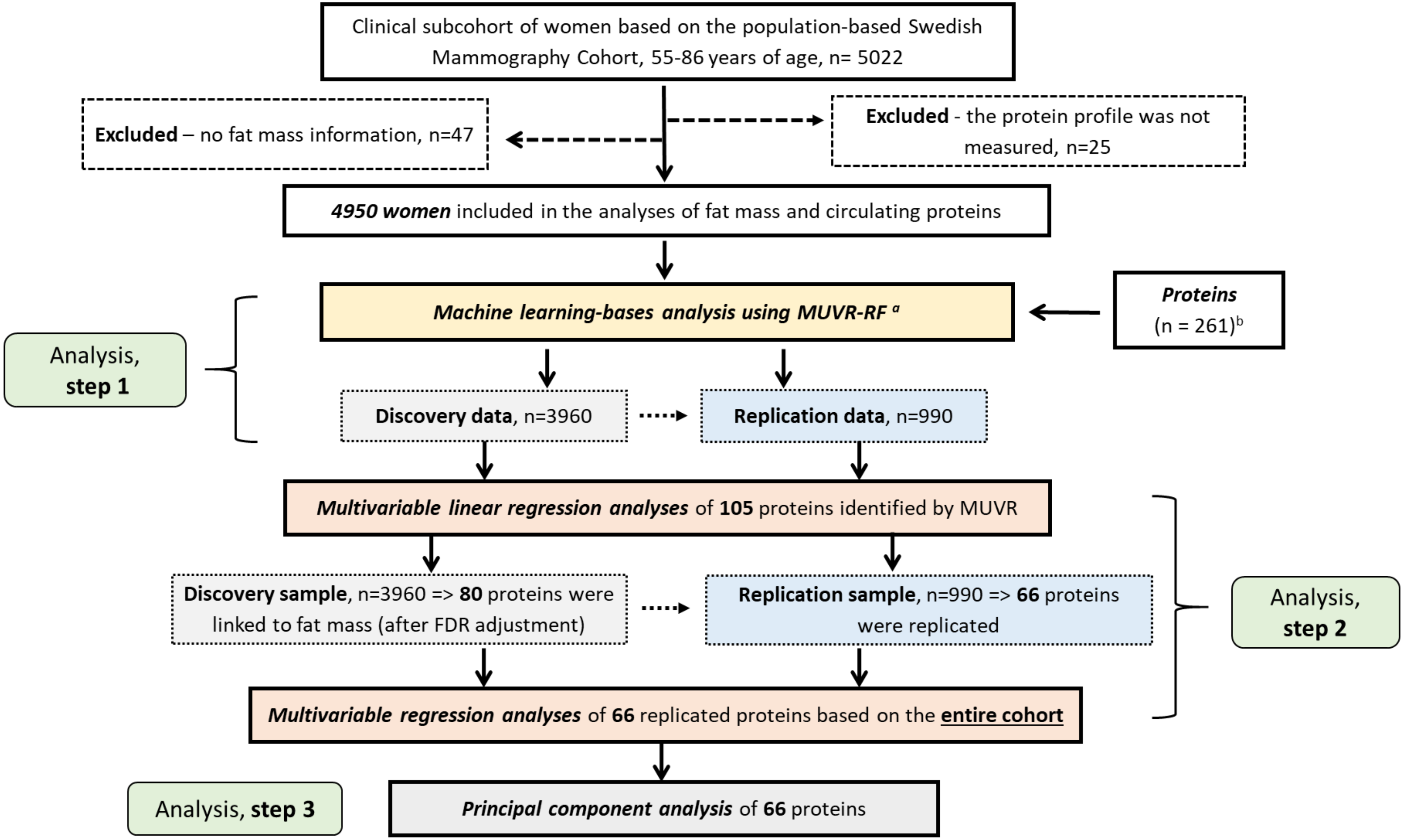

Supplementary Figure 2. Random Forest predictions of FMI from protein profiles using the MUVR algorithm in the discovery data used for model training (left) and the replication data never used for training (right). Prediction performance (Q2) was high and similar between discovery and validation data, suggesting strong FMI-proteome associations as well as the absence of overfitting or bias during model training using the MUVR-random forest algorithm.

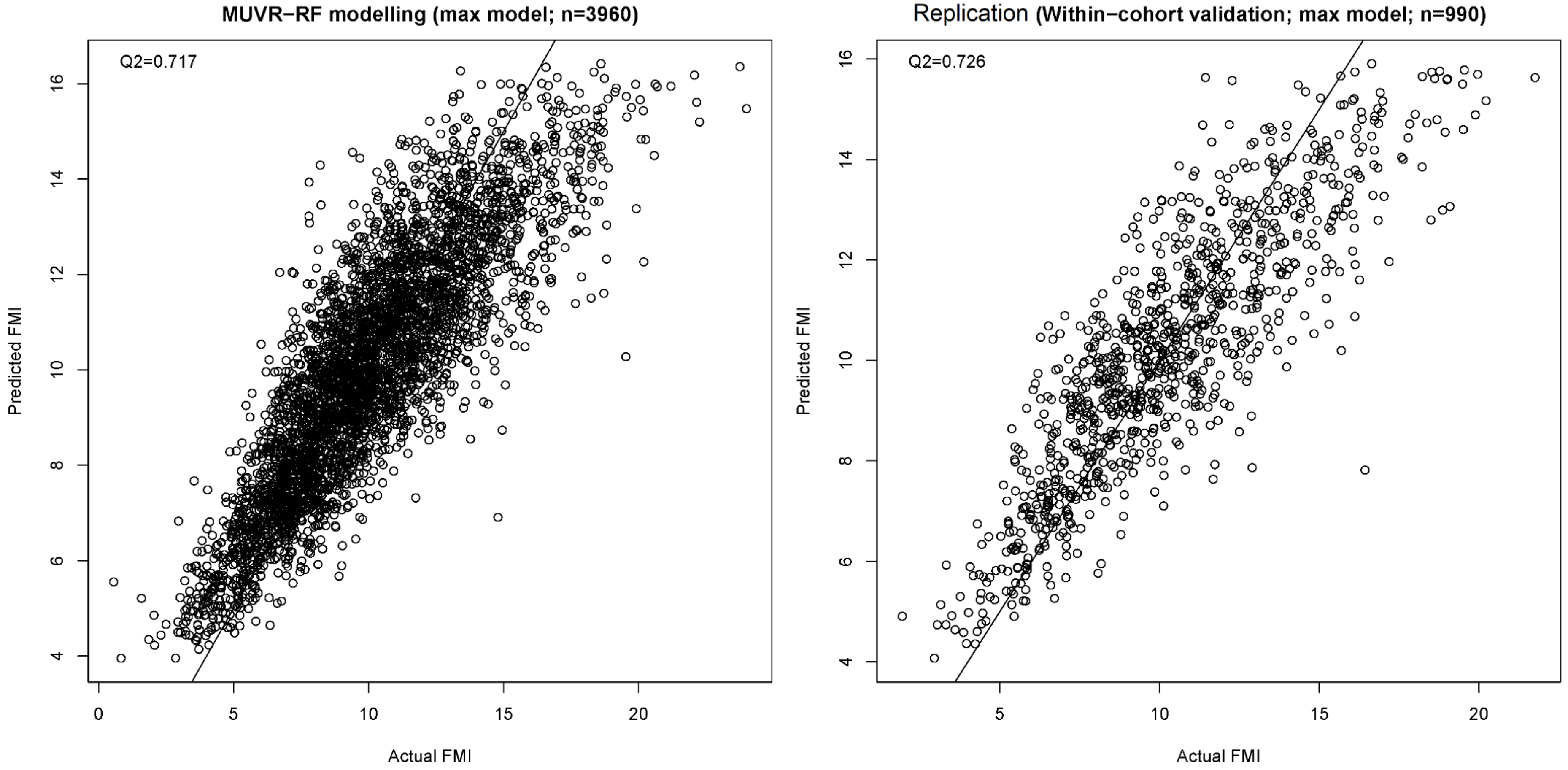

Supplementary Figure 3. Correlation matrix of 105 protein biomarkers identified by the Multivariate methods with Unbiased Variable selection (MUVR).

The color corresponds the correlation coefficient ranging from 1 (dark red) to -1 (dark blue). The complete names of the abbreviated proteins can be found in Supplemental table 1. The proteins were ordered by hierarchical clustering.

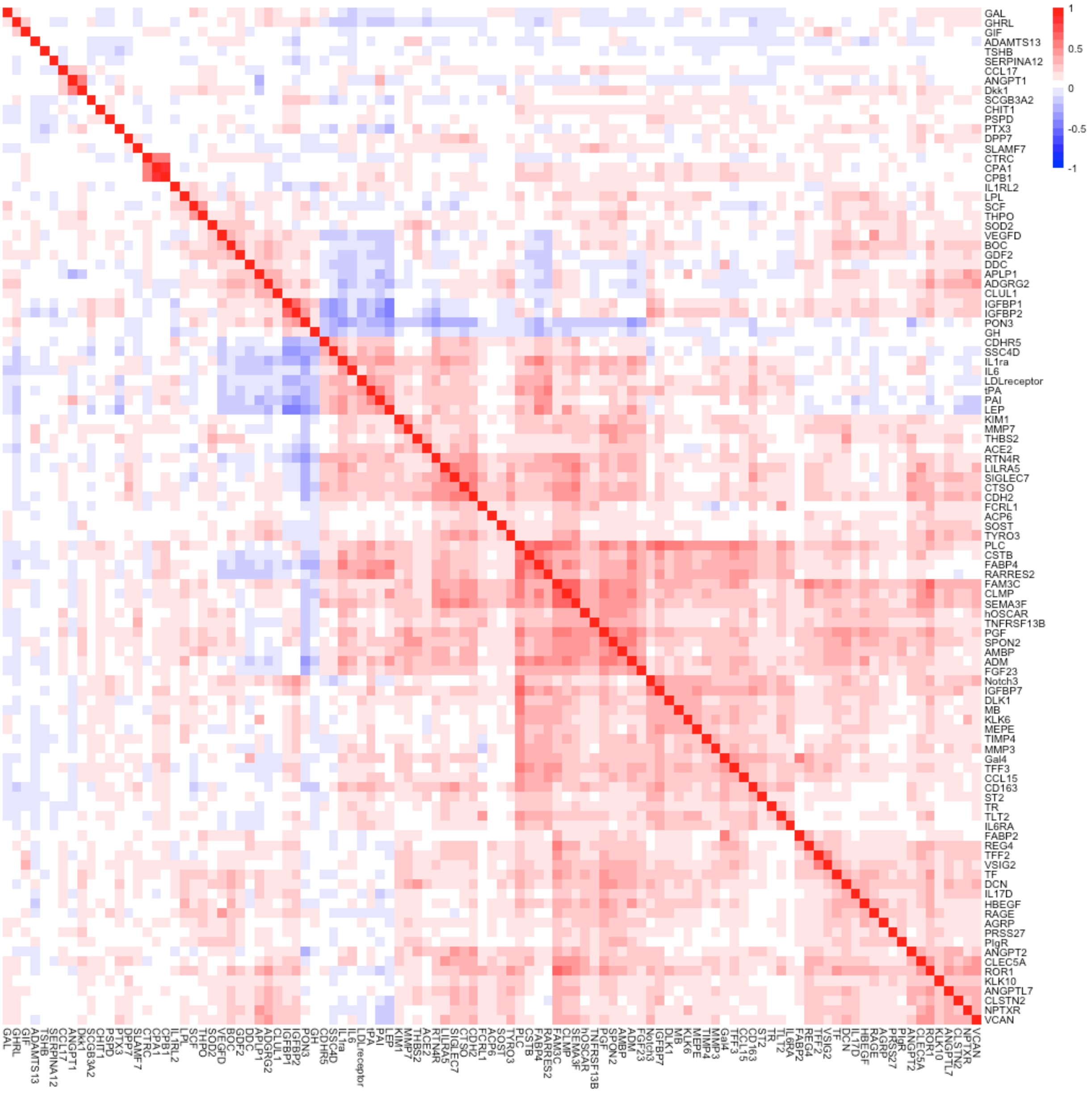

Supplementary Figure 4. Association between total (**A**), android (**B**) and gynoid (**C**) fat mass index and circulating protein biomarkers. β-estimates (per 1 standard deviation change in FMI and biomarker concentration) and 95% CI derived from multiple linear regression analyses. The models were adjusted for age, educational attainment, alcohol intake, smoking status, walking/cycling, leisure-time exercise, and lean mass.

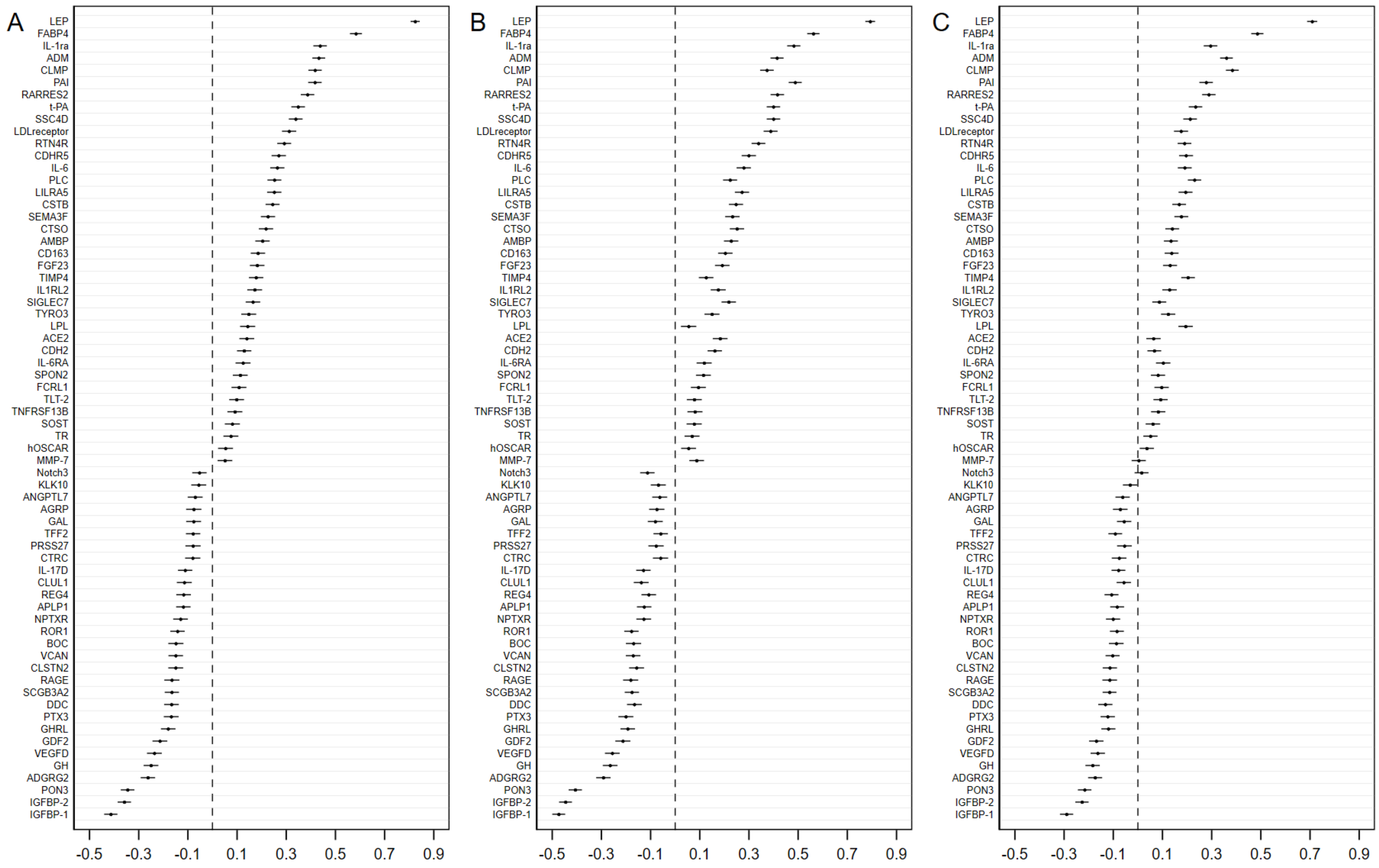

Supplementary Figure 5. Association between *android/gynoid fat mass ratio* and circulating protein biomarkers.

β-estimates (per 1 standard deviation change in biomarker concentration) and 95% CI derived from multiple linear regression analyses. The models were adjusted for age, educational attainment, alcohol intake, smoking status, walking/cycling, leisure-time exercise, and lean mass.

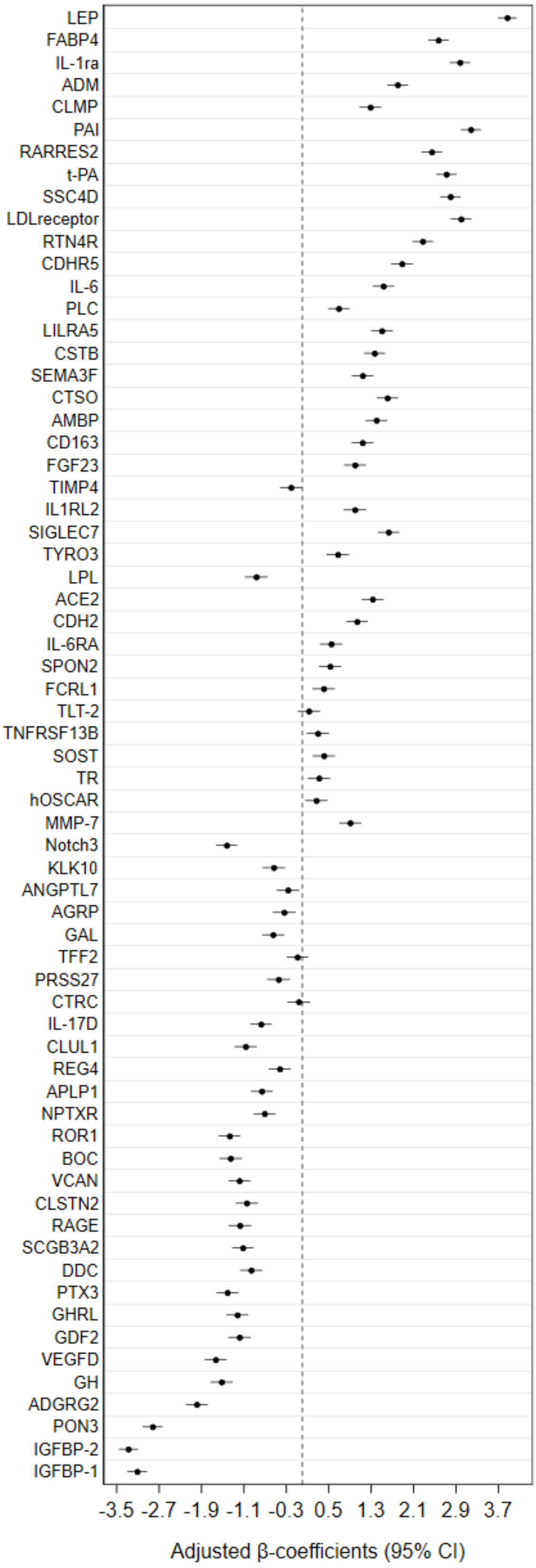

Supplement: Supplementary file 1 — Supplementary material [file 41366_2023_1351_MOESM1_ESM.pdf]
